# Implementation of a novel emergency department pain coach educator program: First year experience and evaluation

**DOI:** 10.21203/rs.3.rs-2488709/v1

**Published:** 2023-01-27

**Authors:** Jennifer H LeLaurin, Magda Montague, Ramzi G Salloum, Sophia S Shiekh, Phyllis Hendry

**Affiliations:** University of Florida; University of Florida College of Medicine; University of Florida College of Medicine; University of Florida College of Medicine; University of Florida College of Medicine

**Keywords:** Pain management, emergency service, implementation science, evaluation, program evaluation

## Abstract

**Background:**

The ongoing opioid epidemic and rising number of patients with chronic pain have highlighted the need for alternative and integrative pain management approaches. A number of evidence-based nonpharmacologic pain management strategies are available; however, these approaches remain underutilized due to barriers such as time limitations, cost, and lack of clinician training. The aim of this work was to implement a nonpharmacologic pain coach educator program that addresses these barriers. We report an evaluation of the first year of program implementation in the emergency department of a large safety-net hospital.

**Methods:**

We implemented a multimodal pain coach educator program that included education on pain neuroscience and over-the-counter analgesic options, demonstration of integrative techniques, and nonpharmacologic toolkits for home use. Implementation strategies included electronic health record tools, training and promotion, clinical champions, and clinician recognition. We used the RE-AIM framework to guide evaluation of the first year of program implementation using data from the electronic health record, quantitative and qualitative program records, and patient-reported outcomes.

**Results:**

In the first year of program implementation 550 pain coach educator sessions were conducted. Upon immediate session completion, 61% of patients felt the program was helpful, 39% were unsure at the time, and none reported session was not helpful. Clinician feedback was overwhelmingly positive. Program cost per patient was $344.35. Adaptations to first year intervention and implementation strategies included modifications of session delivery timing for accommodation of clinical workflows, additions to program content to align with patient characteristics, and changes to patient identification strategies in response to the coronavirus 19 pandemic.

**Conclusions:**

The PAMI pain coach educator program provides a model for nonpharmacologic pain management programs which can be scaled up and adapted for other settings. This work demonstrates the importance of intervention and implementation strategy adaptations to enhance program reach and effectiveness.

## Background

Chronic pain affects more Americans than diabetes, cancer, and heart disease combined and results in an estimated $600 billion in annual costs related to healthcare, disability, and lost productivity (1). Efforts to address the burden of pain in the United States (US), combined with the introduction of new opioid formulations misrepresented as having low potential for abuse, led to widespread misuse of both prescription and nonprescription opioids by the early 2000s (2). This ongoing opioid epidemic in the US has resulted in over 500,000 deaths and cost the economy an estimated $1 trillion in 2017 alone (3, 4). Updated prescribing guidelines along with legislative and regulatory action have corresponded with reductions in opioid prescribing (5, 6); however, patients are increasingly turning to illicit drugs, and opioid-related overdose deaths continue to increase (7, 8). The coronavirus 19 (COVID-19) pandemic has contributed to the rise in opioid-related overdoses and deaths in recent years. Increased inactivity, worsening mental health, increased substance use, and limited access to non-prescription pain management (e.g., physical therapy, medical procedures) can exacerbate pain conditions and lead to a return to opioid use (9).

There is a growing focus in the US healthcare system on delivering nonpharmacologic pain management interventions and reducing opioid use. A number of evidence-based nonpharmacologic approaches to pain management are available, including non-opioid medications, restorative therapies (e.g., physical or occupational therapy), behavioral approaches, and complementary and integrative health (e.g., massage, acupuncture, yoga, tai-chi) (10). Unfortunately, these approaches are often under-utilized in clinical practice or not covered by insurance and many patients lack knowledge of or access to these treatments (10–14).

Safe and effective pain management is particularly challenging in emergency departments (EDs). Pain is the most common reason for ED visits (15, 16). While prescribing rates are declining, about 15% of ED patients receive opioid prescriptions (16, 17). Barriers to effective pain management in ED settings include lack of healthcare professional pain education, lack of access to pain specialists and consistent primary care, time and workflow limitations, insufficient insurance coverage, inadequate patient knowledge about medication safety, and frustration and anger over “shuffling” of care with no improvement in pain (13, 18–24).

The Pain Assessment & Management Initiative (PAMI) was established in 2014 and aims to advance multimodal, safe pain management in healthcare systems to improve outcomes and reduce opioid risk (25). One component of PAMI is a new model pain coaching program, which incorporates patient education and a patient pain toolkit for use after discharge. This program is the first known ED pain coach education program in the US. This paper reports the experiences and evaluation of the first year of program implementation in the ED of a large safety-net hospital.

## Methods

The pain coach educator program provides patient education on nonpharmacologic, integrative, and over-the-counter analgesic options for pain management, along with a patient nonpharmacologic pain toolkit for use after discharge. Since initial program launch, there has been expansion to inpatient and other settings within the health system. This paper reports on experiences in the pre-implementation period (September - December 2020) and first year of implementation (January - December 2021) in the ED setting. The project is registered with the University of Florida Quality Improvement Project Registry.

### Setting

The PAMI pain coach educator program was implemented in the ED of a large (700 bed), urban, safety-net, not-for-profit hospital with approximately 62,000 in-patient admissions annually, serving populations in Northeast Florida and South Georgia. The ED serves over 70,000 patients annually and is the region’s only Level I Adult and Pediatric Trauma Center. Most ED patients identify as Black (62%), are insured by Medicare or Medicaid (54%) or are self-pay or charity city funded pay (24%).

### Program Description

The pain coach educator program is intended for patients age 14 years and older with acute or chronic pain. Patients with psychosis, suicidal or violent behavior, incarceration, severe uncontrolled pain prior to medication, restrained or immobilized, or critically ill were excluded from the program. Patients were referred to the program by physicians or advanced practice providers through an Electronic Health Record (EHR) paging system, phone call, or verbal request from other healthcare professionals (e.g., nurse, physical therapist, pharmacist). Program staff also monitored the EHR tracking board to identify eligible ED patients. The pain coach educator then reviewed the EHR to assess the patient’s relevant medical history to determine appropriateness for the program. When possible, the pain coach educator conferred with a member of the patient’s healthcare team prior to and following the pain coaching session.

Pain coach educator program components are described in detail in a publicly available toolkit on the PAMI website (26). Briefly, sessions consisted of 1) patient education on basic pain neuroscience and prevention of acute to chronic pain transitions, 2) demonstration of integrative pain management techniques, 3) a review of options to improve pain and quality of life, and 4) provision of nonpharmacologic toolkit items and educational brochures, and 5) a review of appropriate OTC and topical analgesic pain management options. The program was intended to be delivered in a single session; however, it was possible for patients to participate in the program during a later admission, ED visit, or via telephone upon patient request. Coaching sessions were tailored based on an initial assessment, type of pain, and patient characteristics and preferences. Patients were provided with a variety of toolkit item options and educational brochures. Brochures covered 17 topics including OTC oral and topical medications, sleep, diet, and back exercises. Toolkit items included aromatherapy inhalers, stress ball symbolizing a pain management analogy (27), hot/cold gel pack, virtual reality cardboard viewer with suggested free virtual reality apps, wearable acupressure device, pain journal, and a QR code to pain management videos on the PAMI website (25). The pain coach educator customized toolkits for each patient based on their pain assessment, contraindications, patient interest, and patient characteristics (e.g., smart phone access, comorbidities). If the pain coach educator was unavailable, clinical team members could provide patients with toolkit items by accessing a stocked cart located within the ED clinical areas.

### Pain Coach Educator Qualifications and Training

The position description for the pain coach educator specified minimum qualifications of a bachelor’s degree in an appropriate discipline and four years of relevant experience or equivalent. Preferred qualifications included a master’s degree, clinical experience, and professional experience in patient education, pain management, neurobiology, or integrative medicine. In the first year, the program was primarily delivered by a master’s-level pain coach educator with formal training in integrative medicine and experience as an emergency medical responder. A small proportion of sessions (approximately 5%) were delivered by other team members with varied backgrounds (e.g., public health, health education) when the primary pain coach educator was unavailable. Orientation and training of the pain coach educators included 1) training in pain neuroscience education and pain management treatment options; 2) shadowing various pain-related healthcare professionals in the hospital system to understand the organizational structure, work environment, and clinical roles; 3) EHR training; and 4) review of current literature, textbooks, and online learning modules related to integrative and nonpharmacologic pain management.

### Implementation Strategies

Four primary implementation strategies were used to promote uptake of the program in the ED during the first year: 1) EHR system modifications, 2) ongoing training and promotion activities, 3) clinical champions, and 4) clinician recognition. To facilitate referrals, the team worked with the health system EHR team to develop an “auto-page” function, which allowed providers to place EHR referrals tied to a specific patient record. An EHR note template and flowsheet were also developed for the program. PAMI staff delivered 22 clinician training and promotion sessions to 397 clinicians and staff during the first year of implementation. Staff also promoted the program through a continuous on-site presence, rounding in clinical areas, email announcements, and visual cues (e.g., bulletin board, flyers). The team engaged clinical champions in various roles (e.g., nurses, residents, physician assistants, physical therapists, pharmacists) to promote the program, serve as a liaison between the program and clinical staff, and provide feedback and updates on program initiatives. Finally, each month the top program utilizer was recognized on a message board in a high-traffic area of the ED, along with supervisor notification and a certificate in their employee file. Further details on the program implementation and lessons learned are available on the PAMI website (25).

### Evaluation

The Reach, Effectiveness, Adoption, Implementation, and Maintenance (RE-AIM) framework was used to guide evaluation. We chose RE-AIM because it is a well-established evaluation framework with the ability to capture patient, clinician, and setting-level outcomes (28). Selected outcome measures organized by RE-AIM are presented in Table 1. Data sources included the EHR, program records maintained by staff, and patient surveys.

### Data collection

The pain coach educator documented delivery of each session in the EHR using a note template created specifically for the program. The template includes the number of past-year ED visits for the patient, history of opioid use or new opioid prescription, toolkit items given, pain management topics discussed, brochures provided, and any additional session details documented narratively by the pain coach educator. The patient’s last documented pain assessment was auto-populated into the note. The PAMI team also developed a structured data collection form including patient demographics, pain characteristics, opioid risk assessment, pain coaching and education session components (topics coached, toolkit items/brochures provided), patient feedback, challenges experienced during the session, and patient disposition including referrals given at discharge. The pain coach educator completed the structured data collection form following each visit. Separate project coordinators conducted chart reviews to complete the remaining sections of the form upon the patient’s discharge from the ED and performed overall data verification. All data were stored and managed in REDCap.

Beginning in November 2021, a subsample of patients also completed a follow-up telephone survey administered by project coordinators one month after the coaching visit to assess at-home use of the coaching topics and toolkit items and gather general feedback. To identify patients for the follow-up survey, program staff generated a random sequence of patients from the prior month and contacted patients by phone one time until 10% completed the survey.

To evaluate clinician training sessions, a pre/post assessment measuring knowledge of nonpharmacologic toolkit items and nonpharmacologic pain management modalities was administered (see Appendix A). The pain coach educator and program staff also recorded informal feedback on the program obtained through discussions with ED clinicians and staff familiar with the program. Feedback was not audio recorded but staff attempted to record comments verbatim when possible. Program challenges and adaptations were systematically documented in weekly team meetings and described in monthly presentations to the funding agency. These presentations and meeting notes were compiled and reviewed by the program team to produce a final list of challenges and adaptations organized by the Framework for Modification and Adaptations (FRAME) (29). Program costs were identified through the US Bureau of Labor Statistics 2021 National Occupational Employment and Wage Estimates for personnel and program budgetary records for all other costs (30).

### Data Analysis

Descriptive statistics were generated for all quantitative measures. We calculated the proportion of referred patients who received the intervention and compared the characteristics of program recipients to all patients admitted to the ED with a pain-related ICD-10 codes (e.g., headache, migraine, musculoskeletal pain, low back pain, renal colic pain) during the evaluation period. Informal feedback from clinicians was reviewed and summarized; no formal qualitative analysis was conducted because responses were limited to brief statements. Cost per participant was calculated by dividing first-year total expenditures by number of participants. Costs for the pre-implementation period were calculated separately.

## Results

### Reach

Over the first year of the program, 550 sessions were conducted with 549 unique patients. Characteristics of pain coach educator program recipients and all patients admitted with pain-related ICD-10 codes are presented in Table 2. Compared to all ED patients with pain-related diagnostic codes, a greater proportion of pain coach educator program recipients identified as female (65.8% vs. 54.4%) and Black (61.4% vs. 52.5%). Direct referrals accounted for 244 (44.4%) of conducted sessions; of these, 134 (54.9%) were verbal referrals and 110 (45.1%) were via the EHR or manual pages. The remaining sessions (n = 306, 55.6%) were conducted with patients identified by pain coach educators and program staff through the EHR track board.

### Effectiveness

Patient satisfaction immediately after the pain coach educator session (n = 550) and 4-week follow up (n = 11) survey responses are presented in Tables 3 and 4, respectively. Immediately after the session, most (61.1 %) recipients reported the session was helpful, 38.9% were unsure, and no patients responded the session was not helpful. When asked which part of the session they found most helpful, patients most frequently identified both the coaching topics and the toolkit items as the most helpful components. Aromatherapy and the virtual reality viewer were the most commonly reported helpful toolkit items. At four-week follow up, all patients surveyed (n = 11) had used the materials and/or education received and gave high ratings (4 or 5 on a 1–5 scale) for the helpfulness of the program in managing pain at home. The majority (n = 8, 72.7%) of patients used program education and/or toolkit items daily.

### Adoption

Clinician comments about the program were overwhelmingly positive. Clinicians described reductions in pain-related anxiety and improved mood in patients receiving coaching sessions. One ED physician stated, “Everyone that talks to the pain coach seems much happier and uplifted.” An ED pharmacist noted the pain coach educator “has demonstrated a positive influence on the patient’s level of empowerment in managing their pain. In conversing with patients, they express appreciation for [the pain coach educator] educating them on techniques such as mindfulness, distraction, virtual reality, and breathing which reduced their pain-related anxiety.” The pharmacist also noted, “the pain coach is being well received by staff; [they] have done a good job of raising awareness of pain coaching services among staff.” An ED nurse also described positive reactions to the program, but noted it was difficult to get program information to night shift staff due to limited program availability at night.

### Implementation

#### Fidelity

Session details are presented in Table 5. All patients were offered education on at least one pain management topic, which the patient could accept or decline. Patients were coached on the majority of educational topics offered, with rates of educational topics offered but not coached (i.e., declined by patient, insufficient time) ranging from 0.4% (virtual reality) to 10.8% (mind/body techniques). Most toolkits distributed included a stress ball, hot/cold pack, aromatherapy, and the PAMI postcard. No challenges were noted for 332 (60.4%) of sessions. The most frequent challenges related to the acuity of the patient’s medical condition (e.g., nausea/vomiting, lethargy) and pain level (e.g., in too much pain to participate, cognition impaired by medication).

#### Program cost

Estimated program costs during the three-month pre-implementation period were $24,117 ($24,011 for personnel, $106 for reference materials); costs during the implementation period totaled $189,047 ($180,797 for personnel, $8,250 for toolkit items). Personnel included: one full-time master’s degree-level pain coach, one part-time (0.5 FTE) coordinator to assist with data entry, chart reviews, and toolkit item management, and one part-time (0.5 FTE) project manager responsible for day-to-day management, reporting, and administrative tasks. Personnel costs were estimated using Bureau of Labor Statistics mean salary for health education specialists (pain coach educator), project management specialists (project manager), and administrative assistants (project coordinators) in general medical and surgical hospitals with 30% fringe benefits. Cost per patient during the first year (excluding pre-implementation costs) was $344.35.

#### Clinician training effectiveness

Pre- and post-tests were administered at two training sessions to 38 residents. Some residents may have attended both training sessions. Most residents had either improved or no change in scores at post-test (n = 22, 57.9%). The remaining residents showed lower scores at post-test (n = 10, 26.3%). The questions with the most incorrect answers at post-test related to belly breathing (23.7%) and hot/cold gel packs (18.4%).

#### Challenges and adaptations

Table 6 presents program challenges experienced and resulting adaptations to the intervention and implementation strategies. Most modifications occurred during the first quarter of the implementation period.

#### Maintenance

**Figure 1:**
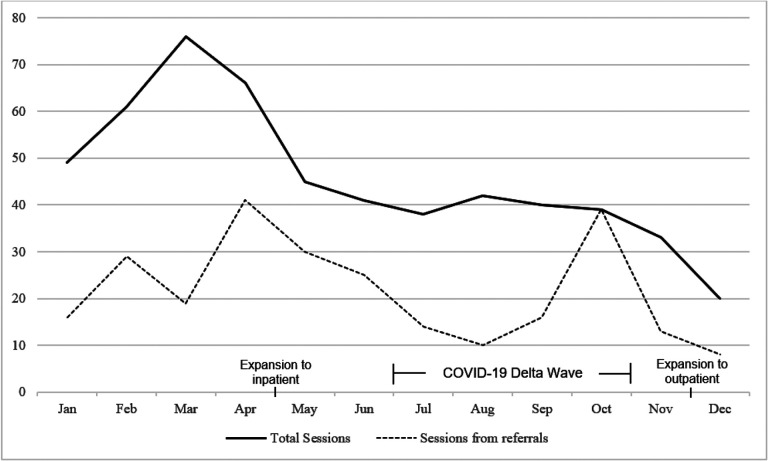
Total sessions and sessions from referral, January - December 2021

The total number of sessions and those resulting from referrals by month are presented in Fig. 1. The number of sessions steadily increased during the first three months of the program and then declined. A decrease in referrals was observed during the COVID-19 Delta variant wave and the related ED overcrowding (31), though total number of sessions did not fluctuate significantly. In the 4-week follow-up survey, 9 (81.8%) patients expressed interest in continuing sessions.

## Discussion

This paper describes the evaluation of the first known pain coach program implemented in the ED. Throughout the first year of implementation, adaptations were made to enhance the fit of the intervention and its implementation with the clinical context, external forces, and patient and clinician needs and preferences. Our findings are promising and support the need for further investigation of program effectiveness and implementation outcomes in the ED and other settings.

In the first year of implementation, 550 ED patients participated in the pain coach educator program. We found program recipients were more likely to be female and black compared to all ED patients with pain-related ICD-10 codes admitted during the implementation period; however, this is an imprecise comparison as diagnostic codes alone are not sufficient to identify patients in need of pain management support (32). Therefore, it is difficult to determine if program recipients were representative of the overall eligible population or if factors known to influence pain management, such as disparities in analgesic administration and influence of culture and gender on pain reporting, played a role (33, 34). In practice, program eligibility assessment relied on clinician consultation and review of both structured and narrative EHR data. In our experience, conferring with clinicians was especially important as use of features such as automated data import or copy-and-paste can result in outdated or inaccurate EHR data (35).

About 60% of patients reported the pain coach educator program was helpful at the conclusion of the session. As the intent of the intervention is to provide education and tools for patients to continue to use after discharge, it is unsurprising that benefits may not be realized immediately for some patients. Further, it is important to note that the remaining patients responded they were unsure of program helpfulness rather than asserting the program was not helpful. While follow-up assessments were newly introduced in the final months of the evaluation period, we found promising results in the small sample that had completed the follow-up questions during year one. All patients had applied the education they received or used toolkit items after discharge and over 70% used the education or items daily. Although our evaluation of effectiveness was limited to assessment of patient satisfaction, previous research shows similar interventions are effective in improving pain intensity, pain interference, pain management self-efficacy, depression, and anxiety (36–38). Notably, one of these studies found a single session program to be as effective as an eight-session cognitive behavioral therapy program (38). In light of this previous work, additional research is needed to determine if the PAMI pain coach educator program produces similar results. In the second year of the program, procedures were implemented to attempt follow-up phone surveys on all patients receiving coaching sessions to better assess program effectiveness. Preliminary data from these assessments show high rates of continued utilization of skills 30 days after the session.

A valuable lesson learned during the first year of implementation was the importance of timing when approaching patients. Patients have high expectations for timely and effective pain management, especially in the ED (39, 40). Shortly after program rollout, the pain coach educator recognized that approaching patients *priorto* analgesic (oral or intravenous) administration was problematic in two ways. First, patients were unreceptive to the program because of their pain and frustration with lack of immediate pain relief. Second, some patients viewed the program as interfering with or replacing analgesic pain management. Modifying session timing to occur after analgesic administration combined with clear communication that participation in the program would not affect receipt of medications allowed for the delivery of more productive sessions to more patients.

Medical education programs typically devote minimal time to pain management and often focus on opioids (41, 42). The PAMI program attempts to address this gap by providing training on nonpharmacologic pain management strategies. Surprisingly, we found that about a quarter of ED residents attending training sessions scored lower on post-test compared to pre-test assessments. Most incorrect answers on post-test assessments were in relation to two topics (belly breathing, hot/cold packs), indicating the need for improved education in these specific areas. Subsequent training sessions focused on clarifying explanation of these topics.

We found the PAMI pain coach educator program cost $344.35 per patient during the implementation period (i.e., excluding start-up costs). Some personnel also supported program expansion to the inpatient setting during the first year of ED implementation, so this cost is likely overestimated. Nonetheless, this cost is relatively low considering the potential for the program to reduce health care utilization and associated expenditures (43). Personnel costs were higher than originally anticipated due to the need for ancillary staff for program management, toolkit inventory, and administrative tasks. Some of this need stemmed from ongoing monitoring and evaluation activities, which could be scaled as appropriate for the implementation setting and resources (e.g., capacity to obtain evaluation metrics from the EHR). These initial findings can be used to inform decisions to implement this and similar programs. Nonpharmacological and integrative pain management approaches have been shown to be cost-effective in treating various types of pain; however, more cost-effectiveness studies specific to pain management in ED settings are needed (44, 45).

The number of monthly pain coach educator referrals and sessions delivered steadily increased in the first three months of program implementation and subsequently declined, which may be attributable to several factors. First, patient eligibility criteria were refined in the first few months of program rollout in response to receipt of referrals for patients who were inappropriate for the program. Second, ED volumes at our institution historically decline in summer and increase in fall and winter. Finally, in late April 2021 the pain coach educator program expanded to include inpatient settings, which reduced program staff availability in the ED. The emergence of the COVID-19 Delta variant corresponded with decreased referrals; however, due to program staff efforts to identify eligible patients, the total sessions per month remained steady during this time. Given the substantial demands on clinician time and attention, particularly in ED settings (46, 47), proactive patient identification by program staff is recommended to supplement direct clinician referral methods when possible.

Strengths of this evaluation include the innovative nature of the pain coach educator program, use of implementation science planning and evaluation frameworks, and systematic documentation of program adaptations. Despite these strengths our approach has some limitations. First, we did not systematically document the number of patients ineligible or uninterested in the program. These procedures have subsequently been implemented in ongoing program activities. Second, clinician feedback was obtained informally rather than through rigorous qualitative methods. Finally, our economic evaluation was restricted to a budgetary impact analysis and did not include effectiveness outcomes.

## Conclusions

This work describes a model for the design and implementation of nonpharmacologic pain management in the ED which can be scaled and adapted for other settings. Our findings reinforce the importance of performing ongoing evaluation and adaptation of interventions and implementation strategies. Future work will evaluate the impact of the program on key outcomes such as admissions and cost-effectiveness in the ED and other clinical settings.

## Figures and Tables

**Table 1:** RE-AIM framework domains and measures

RE-AIM Domain	Measurement	Data Source
**Reach**	Patients receiving intervention	Program records
	Characteristics of PAMI patients vs. overall population of patients with pain-related ICD-10 codes	EHR, program records
**Effectiveness**	Patient satisfaction with coaching sessions	Patient report (post-session)
	Patient use of program skills/materials and program satisfaction	Patient report (4 weeks)
**Adoption**	Clinician feedback	Program records (qualitative)
**Implementation**	Clinician training effectiveness	Training pre/post-tests
	Fidelity (intervention components delivered and session challenges experienced)	Program records
	Program cost	Program records
	Adaptations	Program records
**Maintenance**	Referrals over time	Program records
	Patient interest in future sessions	Patient report (4 weeks)

**Table 2:** Characteristics of program recipients and all emergency department patients with ICD-10 codes for pain, 2021

	Pain coaching recipients (n = 550)		All patients with pain (n = 35,251)^a^	
Characteristic	n	%	n	%
Age (mean, SD)	44.2 (17.0)		48.6 (0.5)	
Gender				
Female	361	65.8	17,566	54.4
Male	186	33.9	14,731	45.6
Other	2	0.4	0	0.0
Race				
Black	337	61.4	18,502	52.5
White	198	36.1	14,446	41.0
Asian	1	0.2	203	0.6
Multiracial	0	0	194	0.6
American Indian	0	0	40	0.1
Pacific Islander	0	0	2	0.0
Unknown	1	0.2	302	0.9
Other	12	2.2	1,562	4.4
Ethnicity				
Not Hispanic	534	97.3	33,387	94.7
Hispanic	15	2.7	1,487	4.2
Unknown	0	0	362	1.0
Area Deprivation Index (mean, SD)	75.0 (22.5)		76.2 (22.3)	
ED final diagnosis				
Abdominal/pelvic pain	103	18.7	--	--
Acute or chronic low back pain	99	18.0	--	--
Arthritis or inflammatory disease	23	4.2	--	--
Cancer related pain	37	6.7	--	--
Headache/Migraine	389	70.9	--	--
Musculoskeletal pain	23	4.2	--	--
Renal Colic	91	16.6	--	--
Sickle cell related pain	9	1.6	--	--
Other	21	3.8	--	--
ED clinician referral reason			--	--
Ongoing pain management	461	85.5	--	--
Patient interested in non-pharmacological/ non-opioid pain management	6	1.1	--	--
Lack of resources	1	0.2	--	--
Other	6	1.1	--	--
None noted	77	14.3	--	--

a Patients with ICD-10 codes for headache, migraine, musculoskeletal pain, low back pain, or renal colic pain

**Table 3:** Patient satisfaction and engagement in coaching topics immediately after pain coach educator session (n = 550)

Survey Item	N	%
Session was helpful		
Yes	335	61.1
No	0	0
Unsure	214	38.9
Most helpful program components		
Education/coaching topics	51	9.3
Toolkit items	21	3.8
Both education/coaching topics and toolkit items	252	45.8
Unsure	203	36.7
Other	18	3.3
No response	6	1.1
Most helpful education topic^1^		
Hot/cold therapy	60	10.9
Aromatherapy	68	12.4
Virtual reality	33	6.0
Diet	8	1.5
Mind/body^2^	102	18.5
Exercises/stretching	22	4.0
Acupressure^3^	15	2.7
Pain education^4^	235	42.7
Other	25	4.5
Most helpful toolkit items^1^		
Aculief^®^	13	2.4
Aromatherapy	135	24.5
Stress ball	41	7.5
Educational materials/brochures	68	12.4
Hot/cold therapy	110	20.0
Pain journal	7	1.3
PAMI postcard	3	0.5
Virtual reality viewer	132	24.0
Other	25	4.5
None	16	2.9
Not applicable	227	41.3

1 Could choose more than one topic or item

2 Includes breathing techniques, mindful meditation, qi-gong, tai chi, and yoga

3 Includes acupressure education and Aculief^®^

4 Includes pain neuroscience education and “Car with Four Flat Tires” analogy”(27)

**Table 4:** Patient satisfaction and engagement in coaching topics at four-week follow-up (n = 11)

Survey Item	N	%
Used materials/education	11	100.0
Methods/materials used		
Aromatherapy	7	63.6
Car with 4 flat tires analogy	7	63.6
Exercise	1	9.1
Hot and cold therapy	7	63.6
Pain Neuroscience Education	1	9.1
Virtual Reality	2	18.2
Other	1	9.1
Helpfulness of pain coaching for pain management at home (1–5)		
4	3	27.3
5	8	72.7
Frequency of techniques, education, or items used		
Daily	8	72.7
Weekly	1	9.1
Never	2	18.2
Would recommend to family or friends		
Yes	9	81.8
No	2	18.2

**Table 5:** Pain Coaching Session Details (n = 550)

	Received		Offered	
Education Topic	n	%	n	%
Pain education^1^	543	98.7	547	99.5
Hot/cold pack	529	96.2	532	96.7
Aromatherapy	502	91.3	510	92.7
Virtual reality	266	48.4	267	48.5
Other	145	26.4	153	27.8
Diet	121	22.0	134	24.4
Mind/body^2^	447	81.3	501	91.1
Exercises/stretching	225	40.9	241	43.8
Acupressure^3^	90	16.4	96	17.5
**Toolkit items given**				
Stress ball	534	97.1		
Hot/cold Pack	527	95.8		
Aromatherapy	505	91.8		
PAMI Post Card	494	89.8		
VR	269	48.9		
Aculief^®^	92	16.7		
Other	10	1.8		
None	4	0.7		
**Challenges experienced**				
None	332	60.4		
Medical condition	64	11.6		
Patient in too much pain	51	9.3		
Time constraints	34	6.2		
Patient not interested	39	7.1		
Patient restrained	11	2.0		
Other	63	11.5		

1 Pain neuroscience education and “Car with Four Flat Tires” analogy(27)

2 Mind/body includes breathing techniques, mindful meditation, qi-gong, tai chi, and yoga

3 Acupressure includes acupressure education and Aculief^®^

**Table 6:** Intervention and implementation adaptations organized by the FRAME (29)

Time (quarter)	Modification category	Description	Goal of modification	Rationale
Q1	Context	Changed session delivery timing to approach patients after analgesic medication administration	Increase reach/engagement	**Patient level:** improve motivation and readiness to participate
Q1	Context	More flexible session delivery, including options to break up sessions	Improve feasibility	**Organization/setting level:** reduce clinical workflow interruptions; work within time constraints and accommodate competing clinical demands
Q1–2	Training	Revised staff and clinician education to clarify program goals and list more explicit eligibility criteria	Improve fit with patients Improve effectiveness Reduce cost	**Provider level:** improve understanding of program and reduce referrals of patients who were inappropriate for the program (e.g., patients with altered mental status)
Q1	Content	Added patient expectation setting component, including clarification the program is an additional service rather than replacement for analgesics	Increase reach/engagement Increase satisfaction	**Patient level:** improve patient receptivity to program and motivation to participate; set realistic expectations for outcomes
Q1–3	Content	Tailored coaching sessions and toolkit materials (e.g., simplified analogies, toolkit items appropriate for patient needs and resources)	Improve fit with recipients	**Patient level:** tailor to patient education/literacy level, available resources, and comorbidities **Organization/setting level:** address communication barriers due to Covid personal protective equipment requirements
Q2–4	Content	Addition of educational topics, toolkit items, and services (e.g., art therapy, pain journal, chaplain service referrals)	Increase satisfaction Improve effectiveness Address cultural factors	**Patient level:** align intervention with patient needs, preferences, and culture
Q3	Implementation and scale-up activities	‘PAMI Star of the Month’ clinician recognition program modeled after existing practices (e.g., resident of the month)	Increase reach/engagement	**Organization/setting level:** align implementation strategies with clinic culture and existing incentives and recognition practices
Q3–4	Implementation and scale-up activities	More proactive identification of patients via EHR by program staff (rather than only clinician referral)	Increase reach/engagement	**Organization/setting level:** compensate for reduced referrals due to increased provider workload and burnout during Covid surges; leverage newly available technology (EHR secure chat) which facilitates communication with providers
Q3–4	Implementation and scale-up activities	Flexible education and promotion delivery (e.g., increased or decreased rounding)	Increase reach/engagement	**Organization/setting level:** reduce activities during Covid surges to accommodate increased workload **Provider level:** increase awareness of program when referrals decrease

## Data Availability

The datasets used and/or analyzed during the current study are available from the corresponding author on reasonable request.

## Abbreviations

ED: emergency department
EHR: electronic health record
FRAME: Framework for Reporting Adaptations and Modifications-Enhanced
PAMI: Pain Assessment and Management Initiative
US: United States
